# Prediction of pregnancy outcome in fresh *in vitro* fertilization/intracytoplasmic sperm injection treatment in patients with poor ovarian reserve

**DOI:** 10.18632/aging.203282

**Published:** 2021-07-16

**Authors:** Ying Chen, AiQin Niu, XingMei Feng, YaLi Zhang, Fei Li

**Affiliations:** 1 Center for Reproductive Medicine, The First People's Hospital of Shangqiu, Shangqiu College of Xuzhou Medical University, Henan, People's Republic of China

**Keywords:** poor ovarian response, pregnancy outcome, POSEIDON, smooth curve fit, live birth

## Abstract

We retrospectively analyzed the clinical data from 39,185 cycles who undergoing *in vitro* fertilization/intracytoplasmic sperm injection (IVF/ICSI) in the First People's Hospital of Shangqiu, these poor ovarian reserve patients were further categorized into the "unexpected" group (n=3337) and the "expected" group (n=2667) based on POSEIDON classification. In "expected" group, logistic regression analysis showed that female age (OR 0.920; 95% C.I 0.902~0.939; P < 0.001), treatment cycles (OR 0.693; 95% C.I 0.560~0.859; P = 0.001), duration of Gn administered (OR 1.077; 95% C.I 1.027~1.129; P = 0.002) and transferable embryos (OR 1.377; 95% C.I 1.319~1.437; P < 0.001) is independent predictive factors of live birth. In "expected" group, logistic regression analysis showed that female age (OR 0.874; 95% C.I 0.848~0.900; P < 0.001), AFC (OR 1.285; 95% C.I 1.131~1.461; P < 0.001), total dosage of Gn administered (OR 1.001; 95% C.I 1.000~1.002; P < 0.001), duration of Gn administered (OR 0.784; 95% C.I 0.639~0.961; P = 0.019), MII number (OR 0.841; 95% C.I 0.717~0.986; P = 0.032) and transferable embryos (OR 2.057; 95% C.I 1.762~2.400; P < 0.001) is independent predictive factors of live birth. We also established a smooth curve fit to predict the probability of live birth among the POSEIDON "unexpected" and "expected" group. These independent predictive factors on the pregnancy outcome of IVF/ICSI and the successful establishment of smooth curve fit can provide valuable reference for treats poor ovarian reserve patients in clinical work.

## INTRODUCTION

How to accurately predict pregnancy outcome in patients with poor ovarian response (POR) remains one of the most elusive medical breakthroughs in humans following assisted reproduction. Despite numerous research efforts, there is no consensus on factors that accurately predict the outcome of *in vitro* fertilization/intracytoplasmic sperm injection (IVF/ICSI) [1, 2]. This has been in part occasioned by poorly understood diagnosis and mechanisms underlying POR development [3, 4], which has frustrated diagnosis and management of infertility among couples. Overall, independent factors that predict pregnancy outcome in POR patients remain to be validated [5].

The new POSEIDON stratification guidelines which classify patients with infertility condition in to "expected" or "unexpected" groups with regard to successful live birth have been embraced by reproductive endocrinologists worldwide. They provide a more nuanced picture for the management of POR individuals in need of reproduction assistance [6, 7]. The POSEIDON concept provides pragmatic clinical recommendations for POR individuals [8]. Live births were based on the number of oocytes needed to obtain one euploid embryo for transfer in each patient [9]. Compared with the Bologna criteria, POSEIDON guides on the diagnosis and management of POR. However, on few researches have been conducted to unravel female-related parameters and independent factors that predict pregnancy outcomes among POR patients, particularly those in POSEIDON clusters. Over the past three decades, several experimental studies have evaluated independent predictive factors for pregnancy outcome following IVF / ICSI in fertile people [10]. Most of the known predictive factors have become part of the routine diagnostic procedure for infertile patients who undergo assisted reproduction. However, comprehensive data specific for POR individuals is either controversial or lacking [11]. The current global aim is to accurately predict the probability of successful pregnancy or live birth in infertile POR patients [12, 13]. This underscores the need to evaluate factors influencing the probability of live birth in POR patients, particularly those in POSEIDON clusters.

Evidence-based medicine has progressively transformed in to the standard approach for numerous diagnosis and treatment of several health complications [14]. Accordingly, we retrospectively analyzed clinical data of 39,185 individuals who underwent IVF/ICSI at the First People's Hospital of Shangqiu, center for reproductive health. We aimed to uncover independent factors for predicting pregnancy outcome among POSEIDON "unexpected" and "expected" group. Findings of this study can improve the management of infertile individual desiring to sire children.

## RESULTS

### Patient characteristics

POR patients were divided in to "unexpected" group (n=3337) and "expected" group (n=2667). Live birth was observed in 1134 (33.98%) women in the "unexpected" group. The rest of the 2203 (66.02%) women didn't get a live birth. In the "expected" group, 220 (8.25%) women delivered live babies, whereas 2447 (91.75%) of them didn't get a live birth.

### Baseline characteristics and independent risk factors for the live birth in the "unexpected" group

**Figure 1:**
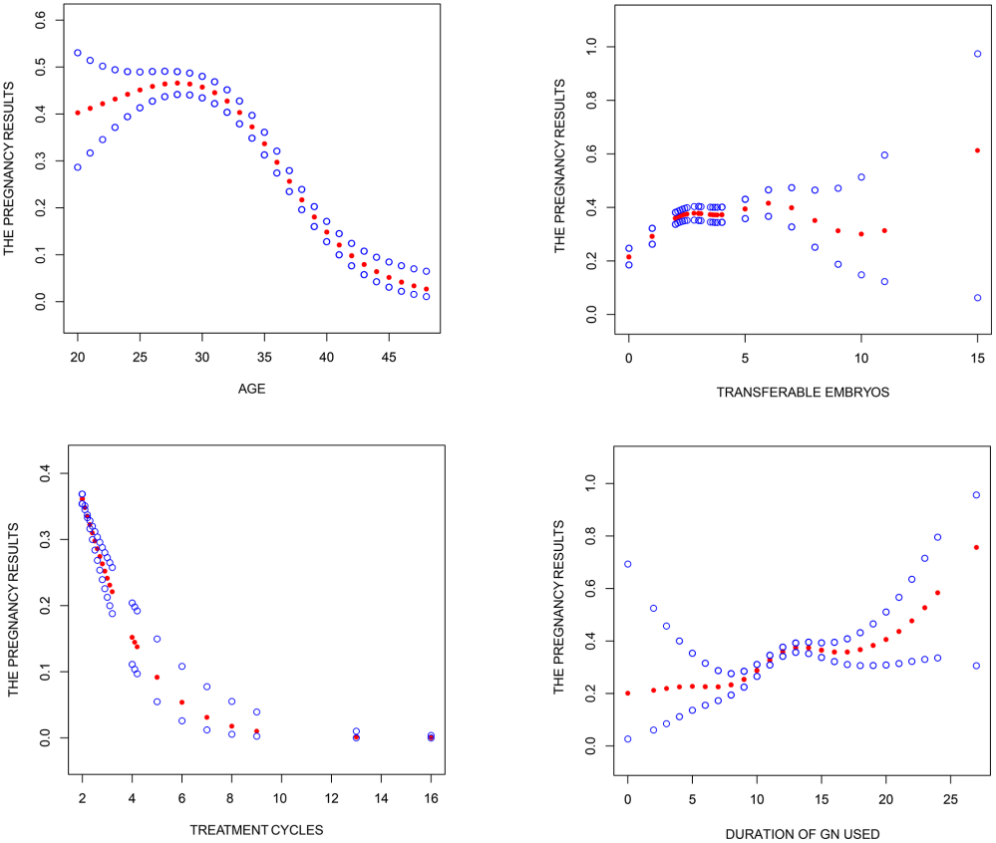
**Association between pregnancy outcome and female age, treatment cycles, duration of Gn used and transferable embryos in "unexpected" group.** A threshold, nonlinear association between pregnancy outcome and these independent predictive factors was found (P<0.05) in a generalized additive model (GAM). Solid rad line represents the smooth curve fit between variables. Blue bands represent the 95% of confidence interval from the fit. Pregnancy outcome was defined as live birth rate; Gn.

Baseline characteristics of patients in the "unexpected" group and likely predictive factors for successful pregnancies are shown in Table 1. Univariate logistic regression analysis revealed that the female age, years of infertility, BMI, basal LH, AMH, AFC, treatment cycles, Gn dosage, duration of Gn administration, number transferable embryos significantly influenced the prospect of live birth. However, multivariate logistic regression analysis showed that the female age (OR 0.920; 95% C.I 0.902~0.939; P < 0.001), number of treatment cycles (OR 0.693; 95% C.I 0.560~0.859; P = 0.001), duration of Gn administration (OR 1.077; 95% C.I 1.027~1.129; P = 0.002) and number transferable embryos (OR 1.377; 95% C.I 1.319~1.437; P < 0.001) were the only independent predictive factors for live birth. Smooth curve fitting showed that female age and treatment cycles increased the risk of unsuccessful births, whereas Gn administration and transferable embryos lowered the risk of unsuccessful births. However, there were no simple linear relationships, threshold effects and nonlinear association were found between female age, number of treatment cycles as well as duration of Gn administration and live birth (Figure 1).

**Table 1:** Baseline characteristics of patients with LB and non-LB and independent risk factors for the pregnancy results in the "unexpected" group.

**Factors**	**Baseline characteristics**		**Unadjusted**			**Adjusted**		
	**LB (1134)**	**non-LB (2203)**	***OR***	**95% C.I**	***P***	***OR***	**95% C.I**	***P***
**Age (years)**	30.9±4.3	33.5±5.6	0.904	0.891~0.918	0.000	0.920	0.902~0.939	0.000
**Infertility years**	4.1±2.9	4.7±3.7	0.939	0.919~0.961	0.000	0.979	0.954~1.006	0.124
**BMI (Kg/M2)**	22.7±3.2	23.0±5.0	0.973	0.951~0.994	0.014	0.989	0.965~1.013	0.361
**Basal FSH (IU/L)**	7.0±2.1	7.0±2.0	1.013	0.979~1.048	0.469	/	/	/
**Basal LH (IU/L)**	5.7±3.5	5.4±3.3	1.026	1.005~1.048	0.015	0.997	0.972~1.022	0.791
**Basal E2 (ng/L)**	40.4±27.9	41.1±29.2	0.999	0.997~1.002	0.464	/	/	/
**Basal P (μg/L)**	0.6±0.7	0.6±0.7	1.042	0.940~1.155	0.437	/	/	/
**AMH (ng/mL)**	3.4±2.8	3.0±2.2	1.065	1.035~1.095	0.000	0.992	0.950~1.037	0.734
**AFC(n)**	13.7±5.8	12.4±5.7	1.037	1.025~1.050	0.000	0.998	0.979~1.017	0.825
**No. of treatment cycles**	2.1±0.3	2.2±0.7	0.570	0.470~0.692	0.000	0.693	0.560~0.859	0.001
**Total dosage of Gn used**	2661.9±994.1	2833.6±956.5	1.000	1.000~1.000	0.000	1.000	1.000~1.000	0.348
**Duration of Gn used**	12.7±2.5	12.3±2.6	1.067	1.038~1.098	0.000	1.077	1.027~1.129	0.002
**Oocyte number**	7.8±2.7	7.9±4.1	0.990	0.971~1.010	0.320	/	/	/
**MII number**	6.3±2.6	6.2±3.7	1.016	0.995~1.038	0.137	/	/	/
**Transferable embryos**	3.5±1.7	2.4±1.9	1.382	1.326~1.441	0.000	1.377	1.319~1.437	0.000

Data are shown as means ± standard deviation. BMI, body mass index; FSH, follicular-stimulating hormone; LH, luteinizing hormone; E2, estradiol; P, progesterone; AMH, anti-Müllerian hormone; AFC, antral follicle counting; LB, live birth; Gn, Gonadotropin.

### Baseline characteristics and independent risk factors for the live birth in the "expected" group

**Figure 2:**
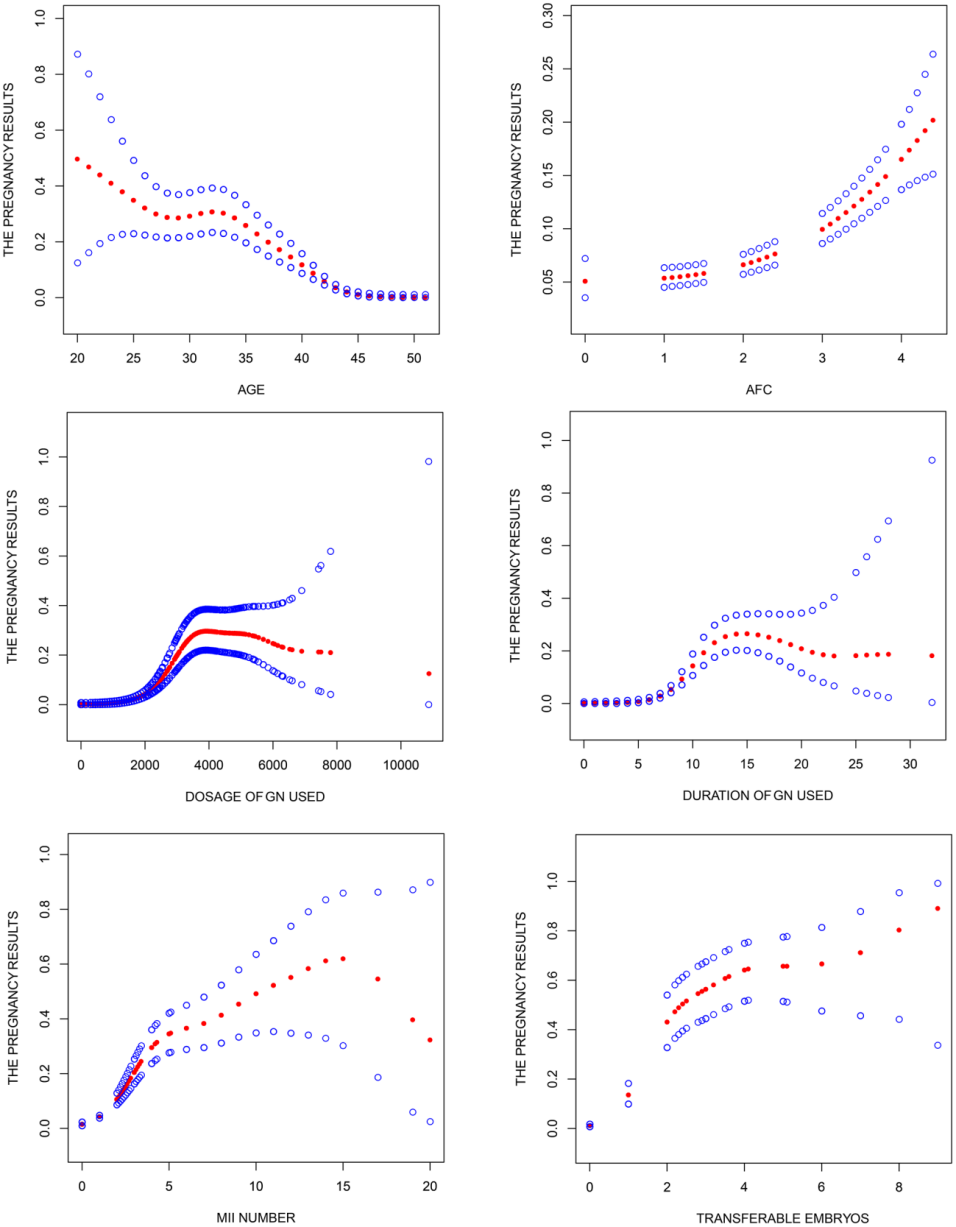
**Association between pregnancy outcome and female age, AFC, total dosage of Gn used, duration of Gn used, MII number and transferable embryos in "expected" group.** A threshold, nonlinear association between pregnancy outcome and these independent predictive factors was found (P<0.05) in a generalized additive model (GAM). Solid rad line represents the smooth curve fit between variables. Blue bands represent the 95% of confidence interval from the fit. Pregnancy outcome was defined as live birth rate, Gn, Gonadotropin; AFC, antral follicle counting.

The baseline characteristics of patients in the "expected" group are shown in Table 2. Univariate logistic regression analysis showed that the female age, years of infertility, basal FSH, basal LH, basal E2, AMH, AFC, number of treatment cycles, total Gn dosage, duration of Gn administration, number of oocytes, MII number and number of transferable embryos significantly influenced the probability of live birth. However, multivariate logistic regression analysis showed that the female age (OR 0.874; 95% C.I 0.848~0.900; P < 0.001), AFC (OR 1.285; 95% C.I 1.131~1.461; P < 0.001), total dosage of Gn administered (OR 1.001; 95% C.I 1.000~1.002; P < 0.001), duration of Gn administration (OR 0.784; 95% C.I 0.639~0.961; P = 0.019), MII number (OR 0.841; 95% C.I 0.717~0.986; P = 0.032) and number of transferable embryos (OR 2.057; 95% C.I 1.762~2.400; P < 0.001) were independent predictive factors for live birth. Smooth curve fitting showed that female age, duration of Gn administered and MII increased the risk of live birth, whereas having antral follicles, total Gn administration and transferable embryos increased the chance of live birth. However, smooth curve fitting showed no substantial nonlinear association between female age, total dosage of Gn administered, duration of Gn administered, MII number as well as transferable embryos and live birth (Figure 2).

**Table 2:** Baseline characteristics of patients with LB and non-LB and independent risk factors for the pregnancy results in the "expected" group.

**Factors**	**Baseline characteristics**		**Unadjusted**			**Adjusted**		
	**LB (220)**	**non-LB (2447)**	***OR***	**95% C.I**	***P***	***OR***	**95% C.I**	***P***
**Age (years)**	34.3±5.0	39.2±5.5	0.869	0.849~0.890	0.000	0.874	0.848~0.900	0.000
**Infertility years**	4.5±3.3	5.3±4.7	0.959	0.927~0.992	0.014	0.986	0.946~1.029	0.528
**BMI (Kg/M2)**	23.2±3.0	23.4±4.3	0.985	0.941~1.032	0.522	/	/	/
**Basal FSH (IU/L)**	9.8±5.2	12.1±6.7	0.933	0.908~0.960	0.000	0.985	0.946~1.026	0.459
**Basal LH (IU/L)**	4.7±2.4	5.9±4.	0.906	0.862~0.953	0.000	0.982	0.922~1.047	0.586
**Basal E2 (ng/L)**	48.6±44.0	58.2±62.5	0.997	0.994~1.000	0.027	0.999	0.995~1.003	0.719
**Basal P (μg/L)**	0.5±0.3	0.5±0.5	0.731	0.499~1.069	0.106	/	/	/
**AMH (ng/mL)**	0.6±0.3	0.4±0.3	8.110	5.255~12.516	0.000	1.183	0.633~2.210	0.599
**AFC(n)**	2.8±1.3	2.2±1.3	1.474	1.310~1.657	0.000	1.285	1.131~1.461	0.000
**No. of treatment cycles**	1.5±1.0	2.2±1.7	0.612	0.523~0.717	0.000	0.912	0.773~1.075	0.271
**Total dosage of Gn used**	3761.8±879.3	2725.2±1400.2	1.001	1.000~1.001	0.000	1.001	1.000~1.002	0.001
**Duration of Gn used**	12.8±2.9	10.0±4.1	1.182	1.141~1.224	0.000	0.784	0.639~0.961	0.019
**Oocyte number**	5.5±3.3	2.5±2.4	1.342	1.286~1.401	0.000	1.084	0.957~1.228	0.203
**MII number**	4.5±2.8	2.0±2.1	1.401	1.331~1.474	0.000	0.841	0.717~0.986	0.032
**Transferable embryos**	2.7±1.4	0.9±1.2	2.138	1.946~2.349	0.000	2.057	1.762~2.400	0.000

Data are shown as means ± standard deviation. BMI, body mass index; FSH, follicular-stimulating hormone; LH, luteinizing hormone; E2, estradiol; P, progesterone; AMH, anti-Müllerian hormone; AFC, antral follicle counting; LB, live birth; Gn, Gonadotropin.

## DISCUSSION

Herein, we analyzed factors that potentially predicts pregnancy outcome after IVF/ICIS among POSEIDON "unexpected" and "expected" groups in POR patients. Assisted reproductive technologies (ART) have significantly improved pregnancy outcomes in POR patients. However, intervening factors for pregnancy outcome in these group of individuals remain uncertain [15]. Also, how POR individuals respond to ART remain unclear. Only few researches have evaluated female-related parameters that independently predict pregnancy outcome in POR patients, particularly those in POSEIDON expected and unexpected clusters. Accordingly, we aimed to bring this to light.

Studies have shown that age of female is the most important factor affecting female fecundity and oocyte quality, all which directly affects pregnancy outcome whether in "unexpected" and "expected" group. Herein, we found a strong relationship between female age and the likelihood of live birth, consistent with previous findings. Particularly age and the propensity of live birth display an inverse relationship. Ovarian function decline with age, which positively correlates with decrease in the number and quality of oocytes [16]. Fewer and weak oocytes lowers the chance of successful pregnancy following IVF/ICSI [17]. Also, monochromatic abnormalities in oocytes and aneuploidy in preimplantation embryos are more common in older women, and are the main causes of miscarriage and lower live birth rates in aged women [16]. Overwhelming evidence shows that reproduction, age-related changes in women such as quantity and quality of oocytes begin at 35. Accordingly, ART live birth rates begin to decline at this age [1, 18]. On the other hand, there is no consensus on the role of FSH and AMH in pregnancy outcome [19, 20]. Herein, we found FSH and AMH levels had no significant effect on pregnancy outcome in both "unexpected" and "expected" POSEIDON clusters. Maseelall et al. also found that AFC is stronger predictor of ovarian response and pregnancy outcome than FSH [13]. and the AFC has been proved to be the best predictor of clinical practice [21, 22]. Studies show that production of more than 6 mature oocytes increases the probability of successful pregnancy by 61.5% [23], consistent with our findings. However, given the selection bias in retrospective studies, randomized controlled trials are needed to validate the relationship between them.

Multivariate logistic regression analysis revealed that age of female, treatment cycles, duration of Gn administered and transferable embryos were independent predictive factors of live birth in the "unexpected" POSEIDON group. our study show that the chances of achieving a successful pregnancy outcome decreases with increasing female age and increasing treatment cycles, however, there is a positive correlation association between the chance of pregnancy and duration of Gn administered and transferable embryos. It is worth emphasizing that there are no simple linear relationship between them observed through smooth curve fit, threshold effects and nonlinear association were found between female age, treatment cycles, duration of Gn administered and live birth, through the integration of large sample data and the smooth curve fit, we can predict the live birth rate more accurately and successfully. And we can found female age maybe in 20 to 30 years had the highest live birth among the "unexpected" group, Meanwhile, the probability of females older than 45 years in the "unexpected" POSEIDON group getting pregnant was generally zero. Also, we found a strong relationship between live birth rate and number of treatment cycles, duration of Gn administration and the number of transferable embryos in POR patients.

Age of female, AFC level, total dosage of Gn administered, duration of Gn administration, number of MII and transferable embryos were independent predictors of live birth in the "expected" group. We found age of female, duration of Gn administration and MII number influences the probability of live birth, whereas AFC, total dosage of Gn administered and number of transferable embryos protective factor for live birth rate. Smooth curve fit revealed that these independent predictors can accurately predict the probability of live birth in POR patients, thus useful in clinical management of these group of individuals. Even so, many factors and functioning of many systems continuous change during pregnancy, coupled by numerous confounding factors. Also, retrospective trials suffer substantial selection bias issues, which impacts on the outcome, we will conduct RCT to confirm this hypothesis in the future.

Overall, the POSEIDON classification system of individuals with infertility complication in to "expected" or "unexpected" groups is useful in clinical diagnosis and management of these patients [24, 25]. This enhances probability of live birth after IVF/ICSI.

## MATERIALS AND METHODS

Clinical data for 39,185 women undergoing IVF/ICSI at reproductive medical center of the First People's Hospital of Shangqiu were enrolled for this study. Eligible participants were categorized into "unexpected" (n=3337) or "expected" group (n=2667) based on POSEIDON criteria. According to POSEIDON guidelines, patients with antral follicle count (AFC) ≥ 5, anti-Mulller hormones(AMH) ≥ 1.2 ng/ml and ≤ 9 oocytes retrieved in the first simulation cycle are (not expected) to have successful deliveries, whereas those with AFC < 5, AMH < 1.2ng/ml are (expected/ not expected) to have successful pregnancies [26, 27].

The patients either received prolonged GnRH agonist or GnRH antagonist treatment, short GnRH agonist treatment or natural cycle of IVF/ICSI followed by embryo transfer in the same cycle. The starting gonadotropin dose was based on the AFC, age of female patient, body mass index (BMI) and previous history of ovarian response to stimulation. The doses were adjusted progressively according to patient`s response. When dominant follicles measuring > 16mm reached 60% or the mean follicle diameter reached 20 mm, (ovulation) triggering was performed using 250ug r-hCG (Merck Serono) in combination with 2000IU u-HCG (Livzon Pharmaceuticals). Oocyte were retrieved 37 hours after triggering using transvaginal ultrasound [28]. Successful pregnancy outcome was defined as at least one baby born alive, or a live fetus born after 28 weeks of gestation [29].

Various parameters and likely intervening factors for successful birth were compared between groups. The data used in this study was downloaded from the Clinical Reproductive Medicine Management System/Electronic Medical Record Cohort Database (CCRM/EMRCD) of the First People's Hospital of Shangqiu, Reproductive Medical Center. The patients had attended the hospital between January 2013 and December 2018. The protocol for this study was approved by the Ethics Committee of Reproductive Medicine Centre of the First People's Hospital of Shangqiu, China. Approved consents were not necessary. The research was performed in accordance with the First People's Hospital of Shangqiu guidelines and regulations.

### Statistical analysis

Data was analyzed using R software, V. 3.2.3, EmpowerStats (http://www.empowerstats.com) and SPSS V. 19.0 (IBM, Armonk, NY, USA). Continuous variables were expressed as means ± standard deviation. Differences between groups were compared using Student’s t test or the Wilcoxon rank sum test. The relationship between various factors such as age of the female, years of infertility, BMI, AMH, AFC, number of transferable embryos, treatment cycles, Gn dosage, Gn duration, number of Oocytes, number of MII and number of transferable embryos among others, and pregnancy outcome was analyzed using univariate and multivariate logistic regression analyses. P < 0.05 was considered statistically significant. P-values were adjusted based on the Holm-Bonferroni method to control for Type I error.
